# Molecular Long-Term Analysis of the GMMG-HD4 Trial in Multiple Myeloma—Patterns of Association of Chromosomal Aberrations with Response and Proliferation Determining Survival in Selecting Treatments in View of Limited Resources in Low- and Middle-Income Countries

**DOI:** 10.3390/ijms25126431

**Published:** 2024-06-11

**Authors:** Anja Seckinger, Hans Salwender, Hans Martin, Christof Scheid, Thomas Hielscher, Uta Bertsch, Manuela Hummel, Anna Jauch, Wolfgang Knauf, Martina Emde-Rajaratnam, Susanne Beck, Kai Neben, Jan Dührig, Walter Lindemann, Ingo G. H. Schmidt-Wolf, Mathias Hänel, Igor W. Blau, Katja Weisel, Niels Weinhold, Marc S. Raab, Hartmut Goldschmidt, Mimi Choon-Quinones, Dirk Hose

**Affiliations:** 1 Department of Hematology and Immunology, Myeloma Center Brussels & Labor für Myelomforschung, Vrije Universiteit Brussel (VUB), 1090 Jette, Belgium; 2 Independent Myeloma Alliance, 8808 Pfäffikon, SZ, Switzerland; 3 Department of Internal Medicine II, Asklepios Klinik Altona, 22763 Hamburg, Germany; 4 Department of Medicine, Hematology/Oncology, Goethe-University of Frankfurt, 60590 Frankfurt, Germany; 5 Department I of Internal Medicine, University of Cologne, 50923 Köln, Germany; 6 Abteilung für Biostatistik, Deutsches Krebsforschungszentrum, 69120 Heidelberg, Germany; 7 Medizinische Klinik V, Universitätsklinikum Heidelberg, 69120 Heidelberg, Germany; 8 Institut für Humangenetik, Universität Heidelberg, 69120 Heidelberg, Germany; 9 Onkologische Gemeinschaftspraxis, Agaplesion Bethanien Krankenhaus, 60389 Frankfurt, Germany; 10 Klinikum Mittelbaden, Medizinische Klinik 2, 76530 Baden-Baden, Germany; 11 Katholisches Krankenhaus Hagen, 58099 Hagen, Germany; 12 Department of Hematology, University Hospital Essen, 45147 Essen, Germany; 13 Department of Integrated Oncology, CIO Bonn, University of Bonn, 53127 Bonn, Germany; 14 Department of Internal Medicine III, Klinikum Chemnitz GmbH, 09113 Chemnitz, Germany; 15 Medical Clinic III Hematology and Oncology, Charité University Medicine Berlin, 13353 Berlin, Germany; 16 Department of Oncology, Hematology and Bone Marrow Transplantation with Section of Pneumology, University Medical Center Hamburg-Eppendorf, 20246 Hamburg, Germany; 17 Nationales Centrum für Tumorerkrankungen, 69120 Heidelberg, Germany

**Keywords:** multiple myeloma, response, survival, proliferation, molecular profiling, LMIC

## Abstract

Based on the lack of differences in progression-free and overall survival after a median follow-up of 93 months in our HOVON-65/GMMG-HD4 trial (German part; *n* = 395) randomizing VAD induction (vincristin/adriamycin/dexamthasone)/tandem-transplantation/thalidomide-maintenance vs. PAD induction (bortezomib/adriamycin/dexamethasone)/tandem transplantation/bortezomib maintenance, we discern how chromosomal aberrations determine long-term prognosis by different patterns of association with proliferation and treatment-dependent response, whether responses achieved by different regimens are equal regarding prognosis, and whether subpopulations of patients could be defined as treatable without upfront “novel agents” in cases of limited resources, e.g., in low- or middle-income countries. Serum parameters and risk factors were assessed in 395 patients. CD138-purified plasma cells were subjected to fluorescence in situ hybridization (*n* = 354) and gene expression profiling (*n* = 204). We found chromosomal aberrations to be associated in four patterns with survival, proliferation, and response: deletion (del) del17p13, del8p21, del13q14, (gain) 1q21+, and translocation t(4;14) (all adverse) associate with higher proliferation. Of these, del17p is associated with an *adverse* response (pattern 1), and 1q21+, t(4;14), and del13q14 with a treatment-dependent *better* response (pattern 2). Hyperdiploidy associates with lower proliferation without impacting response or survival (pattern 3). Translocation t(11;14) has no association with survival but a treatment-dependent adverse response (pattern 4). Significantly fewer patients reach a near-complete response or better with “conventional” (VAD) vs. bortezomib-based treatment after induction or high-dose melphalan. These patients, however, show significantly *better* median progression-free and overall survival. Molecularly, patients responding to the two regimens differ in gene expression, indicating distinct biological properties of the responding myeloma cells. Patients with normal renal function (89.4%), low cytogenetic risk (72.5%), or low proliferation rate (37.9%) neither benefit in progression-free nor overall survival from bortezomib-based upfront treatment. We conclude that response level, the treatment by which it is achieved, and molecular background determine long-term prognosis. Chromosomal aberrations are associated in four patterns with proliferation and treatment-dependent responses. Associations with faster and deeper responses can be deceptive in the case of prognostically adverse aberrations 1q21+ and t(4;14). Far from advocating a return to “outdated” treatments, if resources do not permit state-of-the-art-treatment, normal renal function and/or molecular profiling identifies patient subpopulations doing well without upfront “novel agents”.

## 1. Introduction

Multiple myeloma (MM) is a malignant hematological disease characterized by accumulation of clonal plasma cells in the bone marrow. Clinical signs and symptoms relate to the displacement of normal hematopoiesis, generation of osteolytic bone disease, and renal impairment [1]. Treatment is initiated if such end-organ damage is present or is to be evaluated if its occurrence is imminent, as predicted by biomarkers [2,3]. Treatment has significantly improved during the preceding four decades due to the introduction of small molecules and immune-oncological drugs into clinical practice, including monoclonal antibodies targeting CD38 (daratumumab, isatuximab) [4,5] or SLAMF7/CS1 (elotuzumab) [6]. MM is treated by combination treatment whenever possible [7,8]: effective quadruple combinations like the combination of daratumumab or isatuximab with bortezomib, lenalidomide, and dexamethasone (Dara-VRd) followed by autologous stem cell transplantation (ASCT) (e.g., GRIFFIN trial) increases response rates from about 1/3 for single agents [9,10,11,12,13,14,15] to almost 100% of patients [16], becoming the current standard of care. Immune-oncological therapies in development or recently approved, i.e., bispecific antibodies or CAR-T cells against, e.g., BCMA, GPRC5D, or FCR5H, show single-agent remission rates of 60–80% [17,18,19,20,21,22,23,24,25,26,27,28]. Early clinical trial results of inclusion of these treatment modalities with Dara-VRd or its modifications [26,29] suggest this to evolve as future standard of care.

However, these options are unavailable for the vast majority of patients in low- and middle-income countries (LMIC) due to a lack of reimbursement and coverage by health insurance.

In our prospective GMMG-HD4/HOVON-65 phase 3 trial for patients with newly diagnosed myeloma [30,31,32], we randomized VAD (vincristine, adriamycin, dexamethasone) induction followed by ASCT and thalidomide maintenance (VAD arm) vs. PAD induction (bortezomib, adriamycin, dexamethasone) followed by ASCT and bortezomib maintenance treatment (PAD arm). At a median follow-up of 93 months, we found a surprising result: whereas progression-free survival (PFS) remained significantly prolonged in the PAD vs. the VAD arm (hazard ratio [HR] = 0.76, 95% confidence intervals [CI] 0.65–0.89, *p* = 0.001), overall survival (OS) had become similar (HR = 0.89, 95%CI 0.74–1.08, *p* = 0.24) [32]. At the same time, bortezomib-based regimens abrogated risk for patients with renal impairment and an adverse molecular background, especially in the presence of del17p13 [31,32,33].

We first address in this paper how mechanistically chromosomal aberrations determine long-term prognosis by different patterns of association with treatment-dependent response and proliferation, including whether responses achieved by different treatment regimens are equal regarding prognosis. Secondly, we determine whether, by molecular profiling and serum parameters, a subpopulation of patients can be discerned for which, e.g., in a situation of economic constraints, as in LMIC, treatments, without upfront “novel” agents would not cause harm, as opposed to those for which such a treatment would need to be seen as mandatory.

## 2. Results

### 2.1. Prognostic Factors for Long-Term Survival—Response, Proliferation, and Molecular Alterations

**Figure 1:**
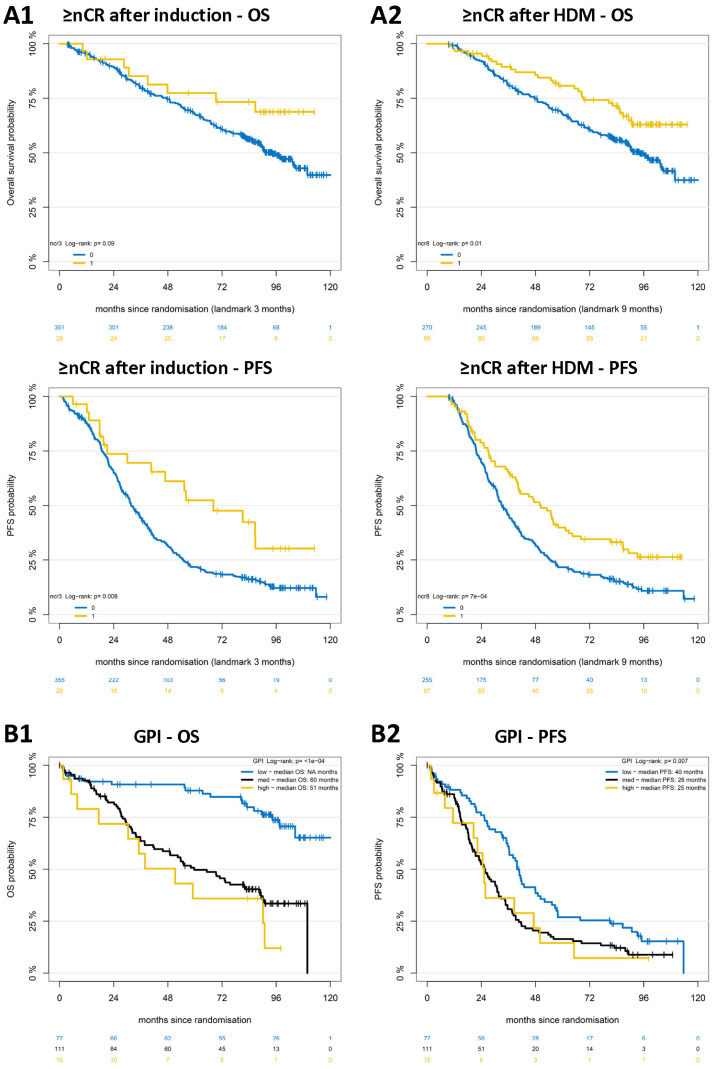
Response and proliferation determine long-term survival. (**A**) Depth of response determines long-term progression-free (PFS) and overall survival (OS) after (**A1**) induction treatment and (**A2**) high-dose melphalan followed by autologous stem cell transplantation (HDM). (**B**) Patients with slowly proliferating myeloma cells show better long-term OS (**B1**) and PFS (**B2**). Blue curve—low proliferation rate (GPI), black curve—intermediate proliferation rate, yellow curve—high proliferation rate. Depicted in each panel is reaching near complete remission or better with ≥nCR = 1, and <nCR = 0.

**Response to treatment.** Reaching near complete remission or better (≥nCR) after induction treatment and HDM followed by ASCT significantly determines long-term PFS and OS (Figure 1A). PFS is 68 vs. 32 months if ≥nCR is reached vs. not reached after induction treatment (*p* = 0.008) and 50 vs. 33 months if reached vs. not reached after HDM (*p* < 0.001). OS is NA vs. 94 months if ≥nCR is reached (not reached) after induction treatment (*p* = 0.09) and NA vs. 94 months if reached (not reached) after HDM (*p* = 0.01; Figure 1A).

**Proliferation** of malignant plasma cells assessed by our gene expression-based proliferation index (GPI) is a strong prognostic factor for OS (*p* < 0.001) and PFS (*p* = 0.007), especially for good long-term survival: patients with GPI^low^ show 74% survival at 8 years (Figure 1B) and do not reach median survival vs. 60 and 51 months in case of GPI^medium^ and GPI^high^, respectively. Median PFS is 40 vs. 25 and 26 months, with 15% of patients not showing disease progression after 8 years (Figure 1B).

The **chromosomal aberrations** del17p13, 1q21+, t(4;14), and del13q14 are associated with significantly adverse PFS and OS, and del8p21 with adverse PFS only. 1q21+ is copy number-dependent associated with adverse survival (2 vs. 3 vs. >3 copies). t(14;16), t(11;14), or hyperdiploidy are not associated with PFS or OS. No aberration investigated is significantly associated with better survival.

### 2.2. Chromosomal Aberrations Determine Long-Term Prognosis by Different Patterns of Association with Proliferation and Treatment-Dependent Response

**Figure 2:**
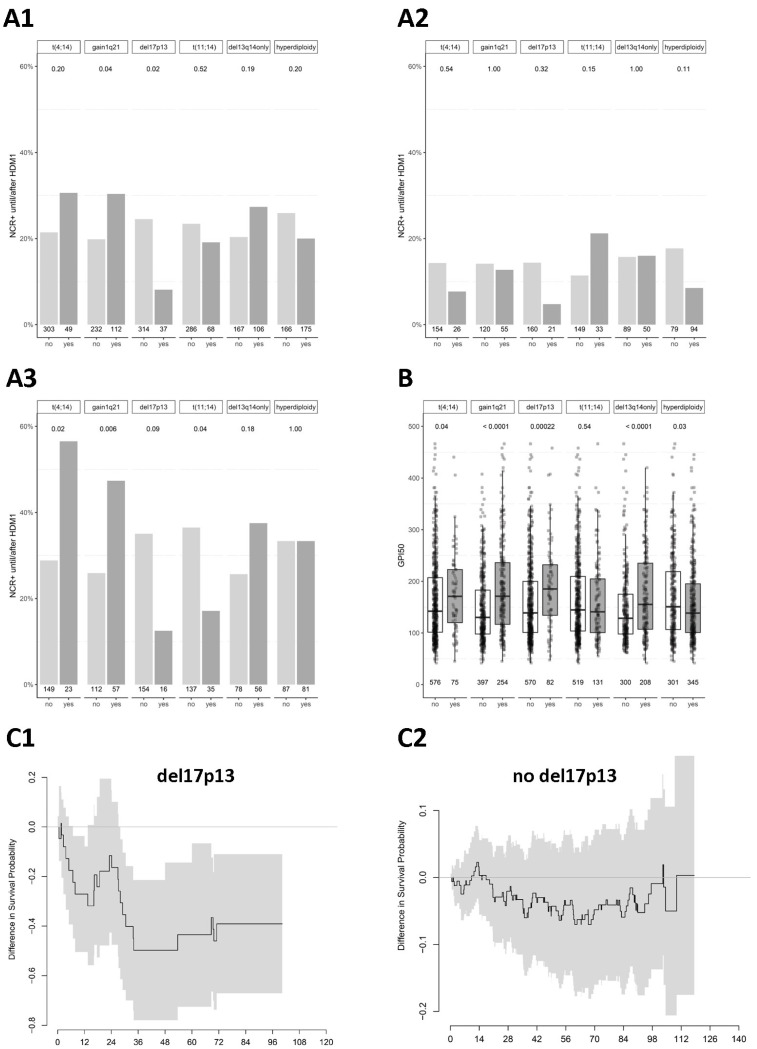

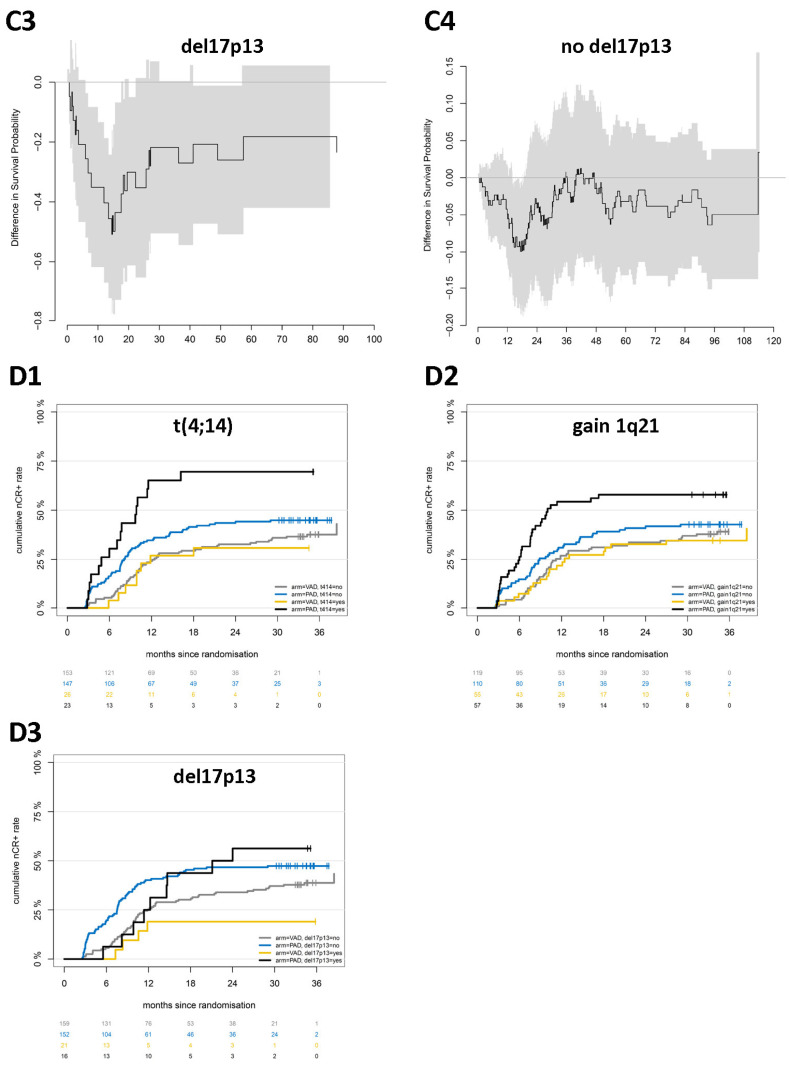
Chromosomal aberrations impact survival by association with proliferation and response. (**A**) Association of chromosomal aberrations with responses (≥near complete response [NCR+]) for (**A1**) all patients, (**A2**) arm A, and (**A3**), arm B, respectively. (**B**) Association of chromosomal aberrations with proliferation (GPI). At the bottom of the figure, the upper row depicts the number of patients with the respective aberration. (**C**) Differences in survival probabilities and association with median time to best response (≥nCR). Differences in survival probabilities for (**C1**,**C3**) patients presenting with del17p13 or (**C2**,**C4**) without. Shown are OS (**C1**,**C2**) and PFS (**C3**,**C4**). (**D**) Association with the median time to best response for patients with (**D1**) t(4;14), (**D2**) gain 1q21, and (**D3**) del17p13. Figure continued on the next page.

**Association with response.** Chromosomal aberrations are associated in different ways with the depth of response after induction treatment and HDM (Figure 2A–C, Table 1). A better response (i.e., ≥nCR) is found in patients with 1q21+ after HDM (30.4% vs. 19.8%, *p* = 0.04). The effect is only seen in the PAD arm (47.4% vs. 25.9%, *p* = 0.006). If 1q21+ is present, ≥nCR is reached more frequently and earlier (*p* = 0.03). Patients with t(4,14) show a significantly better response after HDM (56.5% vs. 28.9%, *p* = 0.01) in the PAD arm, and ≥nCR is reached earlier (*p* = 0.01). The time to best response is significantly shorter, and a higher proportion of patients reach ≥nCR as best response if del13q14 is present in the PAD arm, whereas the opposite is the case in the VAD arm.

Patients with del17p13 show an adverse response after HDM (≥nCR 8.1% vs. 24.5%, *p* = 0.02), without reaching significance for both treatment arms separately (consider low patient number). Hyperdiploidy shows a significantly lower rate of ≥nCR for VAD-based induction treatment (0% vs. 6%, *p* = 0.02) with a reduction in the differential effect after HDM. Patients with t(11;14) show a significantly worse response (17.1% vs. 36.5%, *p* = 0.04) within the PAD arm after HDM. Proliferation (GPI) does not show an association with ≥nCR frequency (Table 1).

**Figure 3:**
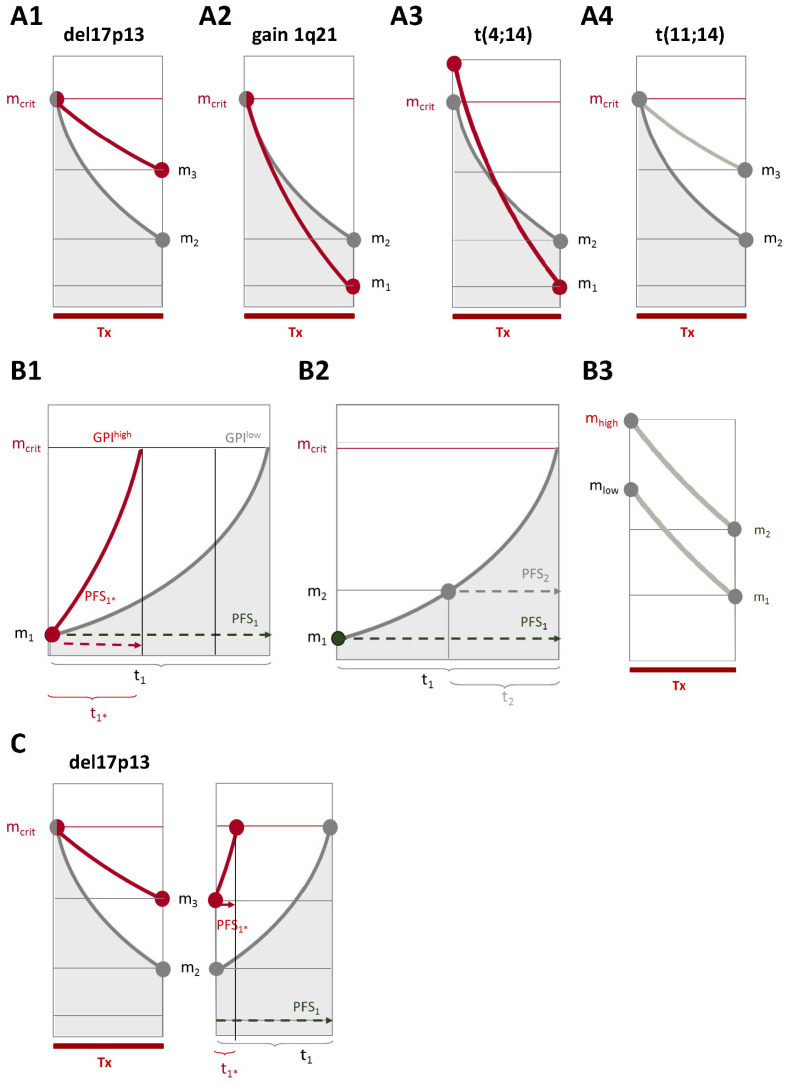
Prognostic impact of chromosomal aberrations, myeloma cell proliferation, and response on patient survival (schematic representation). (**A**) Four patterns of association of chromosomal aberrations with proliferation and depth of remission can be distinguished. (**A1**) Pattern 1: del17p13 (negative prognostic impact) is associated with higher proliferation and adverse response to treatment, leading to higher remaining tumor mass (m_3_). (**A2**) Pattern 2: gain of 1q21 (negative prognostic impact) is associated with a higher proliferation rate but (counterintuitively) a deeper response (lower remaining tumor mass, m_2_, red curve), compared to the absence of the respective aberration (grey curve). (**A3**) t(4;14) is likewise associated with a higher proliferation rate and deeper response (lower remaining tumor mass, m_2_). Additionally, t(4;14) associates significantly with a higher initial tumor mass (red curve). Pattern 3: Hyperdiploidy (prognostically neutral) is associated with a lower proliferation rate without an impact on response. (**A4**) Pattern 4: t(11;14) (no prognostic impact) is associated with a lower proliferation rate but an adverse response. (**B**) Proliferation and response independently impact survival. (**B1**) From an equal response to treatment (remaining tumor mass, m_1_), the faster the proliferation rate (red curve GPI^high^ vs. grey curve GPI^low^), the shorter the PFS. In our data, the chromosomal aberrations del17p13, 1q21+, t(4;14), del13q14, and del8p21 are significantly associated with higher, hyperdiploidy, and t(11;14) with a lower proliferation rate. (**B2**) Impact of remaining tumor mass after treatment on time to progression (TTP) until a critical number of malignant plasma cells (m_crit_) is reached again: the absolute increase in plasma cell number during a given timespan (t) is proportional to the number of plasma cells present at a diagnosis of relapse. For equal proliferation rates, a patient with a better response after treatment (remaining tumor load, m_1_) shows longer PFS (PFS_1_, t_1_) compared to a patient reaching a less deep remission (m_2_, PFS_2_, t_2_). (**B3**) If the same kinetics in cell killing are present, patients with a higher initial tumor mass (m_high_) show a higher remaining tumor mass after treatment (Tx) compared to those with a lower initial tumor mass (m_low_). (**C**) A del17p13 aberration is significantly associated with both less deep remission and faster proliferation, leading to pronouncedly short survival (compare red t_1*_). The lack of remission is partially overcome by PAD-based induction treatment, autologous double transplantation, and bortezomib maintenance, consecutively altering the prognosis and signifying the importance of reaching a deep remission, especially in this subgroup. Lines drawn in red color depict the situation for prognostically adverse factors (e.g., translocation t(4;14) or GPI^high^), and grey-colored lines depict the absence thereof.

**Association with proliferation.** The aberrations t(4;14), 1q21+, del17p13, del13q14, and del8p21 show a significant association with higher proliferation, hyperdiploidy with lower, and t(11;14) shows none (Figure 2D and Figure 3, Table 1).

**Four patterns of association.** Chromosomal aberrations are associated in four patterns with survival, proliferation, and response: del17p13, del8p21, del13q14, 1q21+, and t(4;14) (all adverse) associate with higher proliferation. Of these, del17p13 is associated with an *adverse* response (pattern 1), and 1q21+, t(4;14), and del13q14 with a treatment-dependent *better* response (pattern 2). Hyperdiploidy associates with a lower proliferation rate without impact on response or survival (pattern 3). t(11;14) has no association with survival but a treatment-dependent adverse response (pattern 4). See Figure 3 for a schematic representation.

### 2.3. Responses Achieved by Different Treatment Regimens Are Not Equal

**Figure 4:**
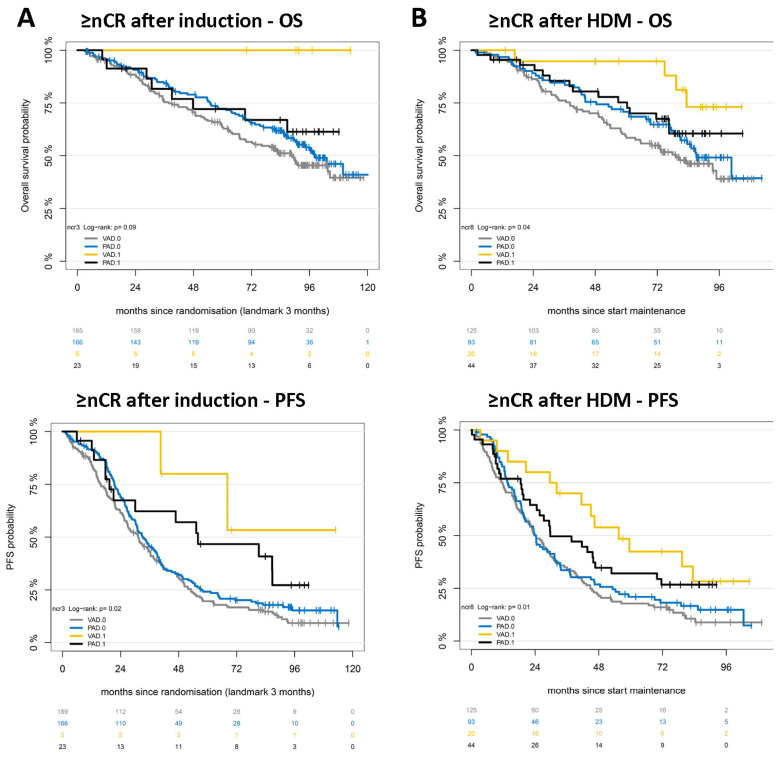
Responses achieved by different treatment regimens are not equal. The best survival is achieved if ≥nCR is reached by VAD induction and high-dose melphalan followed by autologous stem cell transplantation (HDM), but here, the ≥nCR-rate compared to the PAD arm is less than half, i.e., after HDM (13.8% vs. 32.1%) and induction (2.6% vs. 12.1%). The response level (≥nCR) achieved after induction transmits for VAD into better (**A**) overall (OS) and (**B**) progression-free survival (PFS; *p* = 0.046 and *p* = 0.04) unlike PAD induction (*p* = 0.5 and *p* = 0.13, respectively). Responses to different treatment regimens are thus not equivalent in long-term survival. Depicted in each panel is reaching near complete remission or better with ≥nCR = 1, <nCR = 0.

As depicted above (see Figure 1A), the response after induction and HDM (≥nCR, landmark analysis) is prognostic in both treatment arms. The response level (≥nCR) achieved after induction in VAD transmits into better OS and PFS (*p* = 0.046 and *p* = 0.04), unlike PAD induction (*p* = 0.5 and *p* = 0.13, respectively; Figure 2). The best survival is achieved if ≥nCR is reached by VAD induction and thalidomide maintenance. If ≥nCR is reached after induction, the median PFS is NA (arm A) vs. 56 months (arm B), *p* = 0.39, and OS NA (arm A) vs. NA months, respectively, *p* = 0.13 (Figure 4A). If ≥nCR is reached after HDM, the median PFS from the start of maintenance is 55 (arm A) vs. 30 months (arm B), *p* = 0.28, and OS NA (arm A) vs. NA months (arm B), respectively, *p* = 0.17 (Figure 4B). The ≥nCR rate in the VAD arm is however less than half after HDM (13.8% vs. 32.1%) and induction (2.6% vs. 12.1%) of the one in the PAD arm. Responses to different treatment regimens are thus not equivalent in terms of transmission into long-term survival. In other words, not only the level of response matters but also by which treatment regimen it is achieved.

### 2.4. Molecular Background—Specific Gene Expression Patterns for Patients Responding to PAD- vs. VAD-Based Induction

**Figure 5:**
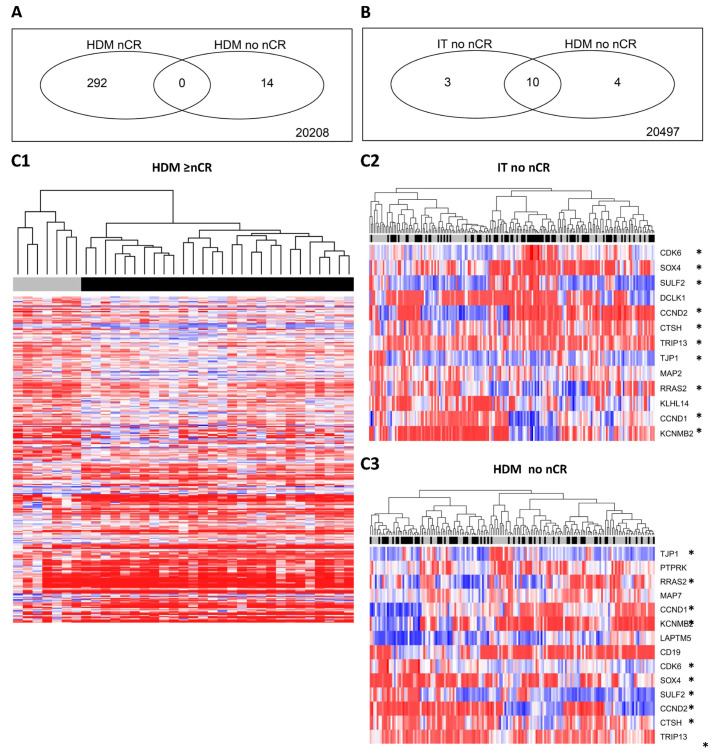
Molecular background of prognostically “unequal” ≥nCR achieved by different treatment algorithms. Significantly fewer patients reach ≥nCR by VAD-based induction alone or VAD and high-dose melphalan (HDM) compared to PAD or PAD plus HDM, respectively. Malignant plasma cells of patients achieving ≥nCR with less active treatment regimens differ significantly in gene expression. (**A**) Comparison of gene expression between patients reaching ≥nCR by PAD vs. VAD-based regimen and HDM (Venn diagram) and overlap with the differentially expressed genes between both algorithms of patients not reaching ≥nCR; 292 vs. 14 genes are differentially expressed (*p* < 0.05, fold change ≥2). (**B**) Comparison of differential gene expression between patients not reaching ≥nCR after VAD vs. PAD induction and HDM, respectively. (**C**) Unsupervised hierarchical clustering (**C1**) based on 292 differentially expressed genes between malignant plasma cells of patients responding with ≥nCR by PAD- vs. VAD-based induction treatment followed by HDM allows delineation of patients responding to either of the treatment arms (gray = VAD; black = PAD). This is not possible for patients not responding with ≥nCR after (**C2**) induction treatment and (**C3**) HDM, respectively. Based on gene expression, the malignant plasma cells of patients responding to each of the regimens show a characteristic pattern of gene expression. For clarity, gene symbols are omitted (see Table S2 for details). Overlapping genes between non-≥nCR responders after induction and HDM, respectively (*n* = 10; see also Figure 4B), are marked with an asterisk.

We next investigated whether specific gene expression patterns of malignant plasma cells can be discerned for patients responding to PAD- vs. VAD-based induction. Comparing patients reaching ≥nCR after PAD-based vs. VAD-based induction and HDM, 292 genes were found differentially expressed (Figure 5A, Table S2), whereas only 14 genes were differentially expressed between patients achieving vs. not achieving at least a ≥nCR, with no overlap of genes between both groups. Considered were genes with a fold change of two or more and significantly different expression (*p* < 0.05). Genes differentially expressed include *DCLK1*, *CDC27*, and *CDKN2C* (CDK-inhibitor P18-INK4C), all with higher expression in the PAD ≥nCR-group, and *ETV1* with higher expression in the VAD ≥nCR-group. Given the low number of patients responding with ≥nCR to VAD induction, the same comparison could not be performed for response after induction treatment. To validate the difference in gene expression, we assessed differences in patients *not* reaching ≥nCR after induction treatment in the PAD vs. VAD arm. As in the comparison after HDM, few (*n* = 13) genes are differentially expressed, ten of which overlap with the genes differentially expressed in the comparison of VAD vs. PAD non-≥nCR responders (see Figure 5B). These genes include *CCND1* and *CD19* (both higher expressed in the PAD non ≥nCR-group) as well as *CCND2* and *CDK6* (higher expressed in the VAD non ≥nCR-group; Figure 5C). Given the time at which the trial was performed, unfortunately, no further molecular analysis was possible.

### 2.5. Who Benefits from the Inclusion of Bortezomib in Upfront Treatment?

**Figure 6:**
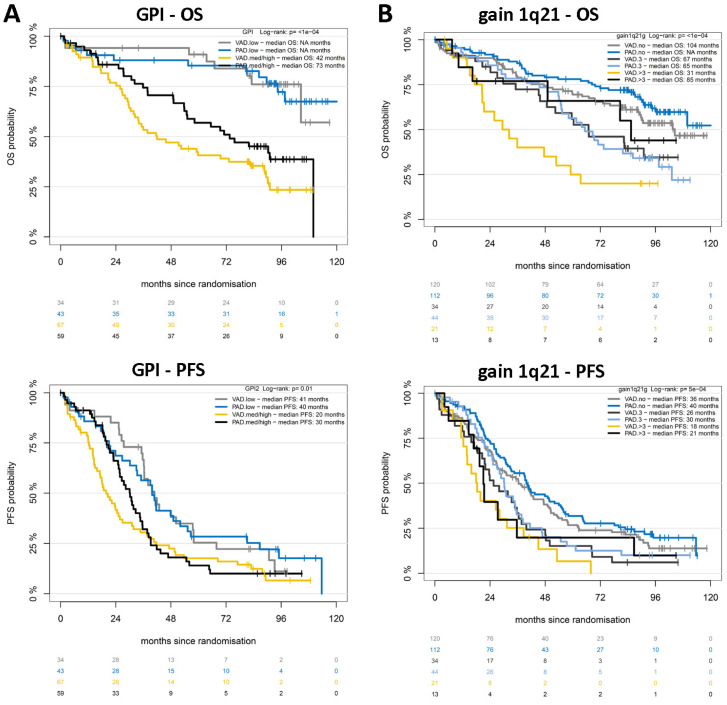
Treatment dependencies. (**A**) For patients with medium/high proliferation, part but not all of the added risk is abrogated regarding progression-free (median PFS; GPI^low^ vs. GPI^med/high^ 41 vs. 20 months (arm A), *p* = 0.015, 40 vs. 30 months (arm B), *p* = 0.051) and overall survival (median OS; GPI^low^ vs. GPI^med/high^ NA vs. 42 months (arm A), NA vs. 73 months (arm B), respectively, *p* < 0.001). For patients with low myeloma cell proliferation, the 8-year PFS and OS are identical between the treatment arms. (**B**) Risk regarding OS for >3 copies of 1q21 compared to 3 copies is likewise abrogated in the PAD arm (median OS for >3 vs. 3 copies, PAD 85 vs. 65 months, VAD 31 vs 67 months).

For patients with medium/high proliferation, part, but not all of the added risk is abrogated (Figure 6A). The same effect is visible for median PFS (GPI^low^ vs. GPI^med/high^, 41 vs. 40 months (arm A), and 20 vs. 30 months (arm B), respectively, *p* = 0.01). As for the absence of high-risk chromosomal aberrations (see below) for patients with low myeloma cell proliferation, the 8-year OS is identical between the treatment arms.

The PAD-based regimen abrogates all or part of the adverse impact of the chromosomal aberrations del17p13 (as previously published [32]) and >3 copies of 1q21 (shown here): For del17p13 (Cox regression model for PFS and OS, interaction *p* = 0.01), the OS hazard ratios (HRs) of del17p13 are 4.4 (95% confidence interval [CI] 2.6–7.3) for the VAD and 1.4 (95%CI 0.7–2.9) for the PAD arm. Similarly, for PFS, the HRs are 3.1 (95% CI 1.9–5.1) for VAD and 1.3 (95% CI 0.7–2.2) for PAD. For patients without del17p13, no difference in survival is observed [32]. We report here that risk regarding OS for >3 copies of 1q21 is likewise abrogated in the PAD arm (HRs (OS)) for >3 vs. 3 VAD arm 1.93 (0.99–3.74), PAD arm 0.77 (0.32–1.87); Figure 6B). No benefit in long-term survival could be shown for the 72.5% of patients with an absence of these aberrations. For the adverse aberrations 1q21+, t(4;14), del13q14, and those being neutral regarding prognosis, i.e., t(11;14) and hyperdiploidy, as well as gene expression-based scores (UAMS GEP70), bortezomib-based treatment prolongs survival both in patients carrying as well as not carrying the respective aberration.

Differences in survival probabilities illustrate how the PAD benefit largely can be attributed to the first 36 months reflecting the on-study duration (Figure 2B). For schematic representation and overview, see Figure 3.

## 3. Discussion

### 3.1. Chromosomal Aberrations Determine Long-Term Prognosis by Different Patterns of Association with Proliferation and Treatment-Dependent Response

Chromosomal aberrations are associated in four patterns with survival, proliferation, and response: del17p13, del8p21, del13q14, 1q21+, and t(4;14) (all adverse) associate with higher proliferation. Faster proliferation is associated with a faster re-emergence of a detectable myeloma clone, i.e., relapse, and adverse survival —a “time-lapsed” myeloma (Figure 3). Of these aberrations, del17p13 is associated with an *adverse* response (pattern 1), and 1q21+, t(4;14), and del13q14 with a treatment-dependent *better* response (pattern 2). Both associations are thus independent and can lead in the same direction (adverse in del17p13 for both treatment arms), or in a different direction (better response but faster re-emergence, 1q21^+^, t(4;14), PAD induction/bortezomib maintenance; neutral regarding VAD-based induction). t(4;14) shows an additional (adverse) association with a higher upfront tumor mass.

Hyperdiploidy associates with a lower proliferation rate without an impact on response or survival (pattern 3). t(11;14) has no association with survival but a treatment-dependent adverse response (pattern 4). Both are in agreement with previous reports [34,35]. Whereas the absolute response rate for t(11;14) is comparable in both arms, the relative response rate for t(11;14) compared to non-t(11;14)-patients is significantly lower in the PAD treatment arm; bortezomib-based treatment works the same as VAD-based induction if a t(11;14) is present, but better if the aberration is not present. This is in agreement with the lack of benefit of t(11;14)-carrying AL amyloidosis patients from bortezomib-based treatment [36]. See Figure 3 for a schematic representation.

Taken together, the level of response is impacted by proliferation (higher = better) but also the molecular subentity, i.e., chromosomal aberration: response is not *a priori* better in case of a higher proliferation rate, e.g., due to eventual higher myeloma cell vulnerability, but is associated with the specific type of chromosomal aberration and the given treatment. Whereas the paradigm adverse response = adverse survival (and vice versa) holds true for the whole patient cohort, it is not true for all molecular subgroups, e.g., t(11;14). A caveat if the response is taken as the endpoint for clinical trials: the divergent behavior regarding the response of different adverse prognostic aberrations suggests avoiding grouping into a single “high-risk-group” and performing an interphase fluorescence in situ hybridization (iFISH) analysis at least for t(4;14), del17p13, and 1q21+, in agreement with the International Myeloma Working Group (IMWG) consensus on risk stratification in multiple myeloma [37] and as included in current updates of the ISS/rISS-score, i.e., R2-ISS [38] and Mayo 2022 score [39].

In our analysis, we likewise show that impaired renal function is associated with the presence of the adverse chromosomal aberrations del17p13, 1q21+, and t(4;14), explaining both parameters losing prognostic significance in the bortezomib-based regimen [32,33].

### 3.2. Responses Achieved by Different Treatment Regimens Are Not Equal

Our trial also answers the clinical question of whether a deep response (≥nCR) with novel agents is “worth” as much as one with “old” agents. This is not the case; patients reaching ≥nCR after VAD/HDM have a significantly better prognosis compared to those doing so after PAD/HDM. We show subsequently that myeloma cells “more easily killed” by a less potent regimen define a biological subgroup. The higher median expression of *CCND1* and *CD19*, both higher-expressed in the PAD non ≥nCR responding group, is in agreement with t(11;14) having a relative (not absolute) lower rate of reaching ≥nCR in this arm compared to the absence of the aberration. In turn, *CCND2* is higher expressed in this group, in agreement with the better response of t(4;14)-carrying patients.

It still has to be proven but likely that this would hold true for a deeper response, i.e., achieving molecular complete remission (minimal residual disease negativity), which was unfortunately not planned to be analyzed in our trial.

### 3.3. Which Patients Profit from Bortezomib-Based Upfront Treatment, and What Lessons Might Be Drawn for Situations of Economic Constraints?

Bortezomib-based upfront treatment abrogates adverse prognosis in patients with del17p13 or renal insufficiency; for 1q21+ and t(4;14), the risk is reduced, as we and others have previously published [31,32,33,40,41]. Here we show that the same amelioration holds true for higher proliferation rates and gene expression-based risk-scores. For the whole trial population, the previously significant difference in OS disappears with longer follow-up, whereas the PFS benefit remains [32]. In addition, we show here that patients with normal renal function (89.4%), low cytogenetic risk (72.5%), or low proliferation rate (37.9%), see Table S1, neither have a benefit in PFS nor OS from bortezomib-based upfront treatment.

What are the potential explanations? First, the difference in survival is largely attributed to the time patients were on the study (Figure 2). Secondly, PFS2 for both treatment arms in long-term follow-up is not different [32]. Thirdly, at the same time, patients with a low proliferation rate or rISS stage I show an excellent long-term survival of approximately 75% at 8 years with both treatment regimens. In patients in whom the treatment worked well, VAD-based induction acted as an additional line of treatment, as bortezomib was available for relapse treatment. In turn, integrating all factors, no patient cohort could be identified profiting from VAD-based instead of bortezomib-based treatment. Given the actual treatment landscape in myeloma, it is naturally not our intention to suggest a “general return of VAD” if state-of-the-art treatment is available. Alas, this is not the case for the vast majority of myeloma patients worldwide, and, in this context, this “last comparison” of old vs. bortezomib-based induction treatment can be taken as a potential guideline to which patients might be considered for “outdated” treatment. Depending on the availability of molecular profiling, different patient populations can be identified neither benefitting in progression-free nor overall survival from bortezomib-based upfront treatment, for which the VAD/HDM/thalidomide maintenance approach might be considered: with a low proliferation rate (37.9%), low cytogenetic risk (72.5%), or normal renal function (89.4%). The latter definition, based on serum creatinine, would be possible in all jurisdictions as a potential “drawback” line.

## 4. Methods

### 4.1. Study Design and Participants

A total of 833 patients (aged 18–65 years) with newly diagnosed MM as defined by the 2003 IMWG criteria [42] (updated in 2014) [2] in the Salmon and Durie stage [43] II–III were enrolled in the prospective, randomized HOVON-65/GMMG-HD4 phase 3 trial (EudraCT no. 2004-000944-26) in 75 centers in the Netherlands, Germany, and Belgium, between May 2005 and May 2008 and followed until 2015 [30,32]. The trial was conducted in accordance with the Declaration of Helsinki (Version 1996) and approved by the ethics committees of the Erasmus University Medical Center, the University of Heidelberg, and all the participating sites. We obtained written informed consent from the patients for treatment and sample procurement. Patients were randomly assigned to arm A (termed VAD arm) or arm B (termed PAD arm). In brief, arm A consisted of 3 cycles of induction treatment with vincristine 0.4 mg intravenously (IV) on days 1–4; doxorubicin 9 mg/m^2^ IV on days 1–4; and dexamethasone 40 mg orally on days 1–4, 9–12, and 17–20. Arm B consisted of bortezomib 1.3 mg/m^2^ IV on days 1, 4, 8, and 11; doxorubicin 9 mg/m^2^ IV on days 1–4; and dexamethasone 40 mg orally on days 1–4, 9–12, and 17–20. Stem cells were mobilized by the use of cyclophosphamide 1000 mg/m^2^ IV on day 1, doxorubicin 15 mg/m^2^ IV on days 1–4, dexamethasone 40 mg orally on days 1–4, and G-CSF (filgrastim 10 µg/kg or lenograstim 300 µg/m^2^) per day subcutaneously divided into 2 doses per day from day 9 until the last stem cell collection. After stem cell collection, patients were treated with 1 or 2 cycles of high-dose melphalan (200 mg/m^2^ IV) (HDM) and ASCT, followed by maintenance treatment with thalidomide (50 mg/d orally in arm A) or bortezomib (1.3 mg/m^2^ IV once every 2 weeks in arm B) for 2 years.

Evaluation of response was performed according to modified European Group for Blood and Marrow Transplantation (EBMT) criteria. Near CR (nCR) and very good partial response (VGPR) were implemented as in the IMWG response criteria [44]. nCR was defined as CR with positive or missing immunofixation [10], and VGPR was defined as more than 90% reduction of serum M-protein and urine light chain less than 100 mg/24 h. CR required negative serum/urine immunofixation and bone marrow morphology evaluation. Responses were assessed after induction, after the first and second transplantation, at 2-month intervals during maintenance, and until progression. PFS was calculated from random assignment until progression, or relapse (as defined by IMWG criteria [44]), or death, whichever came first [30].

For additional information on the trial including, e.g., the CONSORT chart, see Sonneveld et al. 2012 [30].

The German sites decided to perform a comprehensive iFISH analysis as previously published [31]. At study inclusion, bone marrow aspirates from 354 of 395 eligible patients (89.6%), in total, treated at 35 different institutions in Germany were sent to the Labor für Myelomforschung (Multiple Myeloma Research Laboratory) Heidelberg for plasma cell purification and subsequent detection of chromosomal aberrations by iFISH. Follow-up data on PFS and OS were obtained up to 2015. The median OS was 94 months (95%CI: 86–110; 180 events), the median PFS was 33 months (95%CI: 30–36; 310 events), and the median follow-up (time to censoring) was 93 months (95%CI 91–95).

### 4.2. Purification of CD138^+^ Plasma Cells

Density gradient centrifugation of bone marrow aspirates by Ficoll-Hypaque (Biochrom, Berlin, Germany) was performed to separate mononuclear cells by a standard protocol. CD138^+^ plasma cells were isolated using anti-CD138 immunobeads and an autoMACS separation system (Miltenyi Biotec, Bergisch Gladbach, Germany) as published [34,45,46,47]. Purity was assessed using flow cytometry. CD138-purified plasma cell samples were then subjected to iFISH (*n* = 354) and gene expression profiling (*n* = 204); see below and Table S1.

### 4.3. iFISH Analysis

iFISH was accomplished using probes for the detection of numerical aberrations of the chromosome regions 1q21, 5p15/5q35, 6q21, 8p21, 9q34, 11q23, 13q14.3, 15q22, 17p13, 19q13, and 22q11, as well as for the IgH translocations t(11;14)(q13;q32), t(4;14)(p16.3;q32), and t(14;16)(q32;q23) [31,48]. For the detection of numerical aberrations, the following probes were used: CL 1q21/13q14 (MetaSystems (MS) D-5997-100-OG), CL 8p21/19q13 (MS D-5915-100-OG), CL ABL1/SMA (9q34/15q22) (MS D-5961-100-OG), XL ATM/TP53 (11q22.3/17p13) (MS D-5046-100-OG), and NSD1/hTERT (5p15/5q35) (Cytocell, customized). For detection of translocations, the following probes were used: XL IGH Plus Breakapart probe (MS D-5061-100-OG), XL t(11;14) (MS D-5062-100-OG), and only if IGH break positive and t(11;14) negative, XL t(4;14): (MS D-5064-100-OG) and XL t(14;16) (MS D-5072-100-OG).

Hybridization was performed according to the manufacturer’s instructions (Kreatech Amsterdam, The Netherlands; MetaSystems, Altlussheim, Germany; Vysis, Santa Clara, CA, USA). A total of 100 interphase nuclei per probe were evaluated using a DM RXA epifluorescence microscope (Leica, Wetzlar, Germany). Hybridization efficiency was validated on interphase nuclei obtained from the peripheral blood and bone marrow of healthy donors. The thresholds for gains, deletions, and translocations were set at 10%. The score of Wuilleme et al. was used to assess ploidy [49]: gains of at least two of the three chromosomes 5, 9, and 15 define hyperdiploidy.

### 4.4. Gene Expression Analysis

RNA was extracted using the Qiagen AllPrep DNA/RNA kit (Qiagen, Hilden, Germany). Quality control and quantification of total RNA was performed using an Agilent 2100 bioanalyzer (Agilent, Frankfurt, Germany).

Gene expression profiling using U133 2.0 plus arrays (Affymetrix, Santa Clara, CA, USA) was performed as published [34,45,46,47,50,51]. Expression data are deposited in Gene Expression Omnibus and ArrayExpress, respectively, under accession numbers GSE19784 and E-MTAB-2299.

### 4.5. Statistical Analysis

Fisher’s exact test was used to test for associations between categorical variables. The Mann–Whitney Wilcoxon test was used to compare quantitative variables between groups. In the case of ordered factors (e.g., ISS), the Jonckheere–Terpstra test was used. PFS was defined as the time from randomization until progression, relapse, or death, whichever occurred first, but was censored in the case of allogeneic transplantation. OS was calculated from randomization until death from any cause. Patients still alive were censored at the date of last contact. Estimation of PFS and OS distribution was performed by the method of Kaplan and Meier. The log-rank test was used for comparisons of OS and PFS curves. Univariable proportional hazards (PH) regression analysis was used to evaluate the prognostic impact based on HRs including 95% CIs. The interaction term between factor and treatment was tested to assess the heterogeneity of effect. Time to response was defined as the time from randomization to the time of the first response. Patients not reaching response were censored at the time when going off-study. Early drop-out was treated as competing risk. Cumulative incidence curves and Gray’s test [52] were used to compare groups. Response to treatment was defined as best response until/after induction therapy and first high-dose melphalan.

Analysis of gene expression was performed on GC-RMA [53] preprocessed data. Due to two different IVT labeling kits used, batch correction was performed using ComBat [54]. The UAMS GEP70 score (high vs. low risk) [55] as well as the gene expression-based proliferation index (GPI; high vs. medium vs. low risk) [34] were calculated as published. For calculation of the GEP70 score data were normalized using mas5 [56].

The ISS and revised ISS scores (rISS) were calculated as published [57,58].

For associations of molecular and clinical parameters, additional samples from patients included in the GMMG-MM5 trial (EudraCT no. 2010-019173-16) [59] were investigated (*n* = 556 with iFISH and *n* = 458 with gene expression profiling).

Computations were performed using R 3.1.1 (http://www.r-project.org/ accessed on 16 May 2024) and Bioconductor 2.14 [60]. Effects were considered statistically significant if the *p*-value of corresponding statistical tests was <5%.

## 5. Conclusions

Taken together, there are four messages in our manuscript. First, the different prognostic impacts of chromosomal aberrations can be explained by association with proliferation and treatment-dependent better or adverse responses and follow four patterns. Secondly, better response does not mean better survival for all molecular subgroups, as exemplified for 1q21 gain and translocation t(4;14). A caveat for response endpoints in clinical trials: Third, responses to different treatment regimens are not equal in transmission to long-term survival, and thus not worth the same. Underlying is a difference in gene expression of malignant plasma cells already killed by a less active regimen. Fourth, as bortezomib-based upfront treatment abrogates higher proliferation, del17p13, and renal insufficiency, patients without these risk factors do not profit in long-term survival analysis, in case of limited resources such as in LMICs; a comparably less expensive treatment like VAD/HDM/thalidomide maintenance can be seen as an option. In case of a lack of availability of molecular profiling, the choice for upfront treatment might be made based on serum creatinine.

## Figures and Tables

**Table 1:** Association of chromosomal aberrations with proliferation, initial tumor mass, response, progression-free (PFS), and overall survival (OS). If significant, association with higher (↑) or lower (↓) parameters is depicted by an arrow, otherwise by “=”. Positive impact on response, PFS, or OS of the presence of the respective parameter is depicted in green, negative in red, and neutral in grey color. HDM, high-dose melphalan; GPI, gene expression-based proliferation index. Shown also is the percentage of patients who harbor the respective risk factor or absence thereof. CREA ≥ 2 renal impairment (creatinine ≥ 2mg/dL). Pts., patients.

	Pts. [%]	Proli- Feration	Tumor Mass	≥nCR after HDM			CREA ≥2	PFS			OS			Benefit Shown
				ALL	VAD	PAD		ALL	VAD	PAD	ALL	VAD	PAD	
**GPI**	**∕**	**∕**	=	=	=	=		↓	↓	↓	↓	↓	↓	yes
**t(4;14)**	13.9	↑	↑	=	=	↑	↑	↓	↓	↓	↓	↓	↓	yes
**gain 1q21**	32.6	↑	=	↑	=	↑	↑	↓	↓	↓	↓	↓	↓	yes
**del17p13**	10.6	↑	=	↓	=	↓	↑	↓	↓	=	↓	↓	=	yes
**t(11;14)**	19.2	=	=	=	=	↓	=	=	=	=	=	=	=	yes
**del13q14 only**	38.8	↑	=	=	=	=	=	=	=	=	↓	↓	↓	no
**hyperdiploidy**	51.3	↓	=	=	=	=	=	=	=	=	=	=	=	yes

## Data Availability

Expression data are deposited in Gene Expression Omnibus and ArrayExpress, respectively, under accession numbers GSE19784 and E-MTAB-2299.

## Supplementary Materials

The following supporting information can be downloaded at: https://www.mdpi.com/article/10.3390/ijms25126431/s1.
