# Supplementary material for: Molecular Long-Term Analysis of the GMMG-HD4 Trial in Multiple Myeloma—Patterns of Association of Chromosomal Aberrations with Response and Proliferation Determining Survival in Selecting Treatments in View of Limited Resources in Low- and Middle-Income Countries
Source: Int J Mol Sci. 2024 Jun 11;25(12):6431. doi: 10.3390/ijms25126431 (PMC11204152; doi:10.3390/ijms25126431)
Supplement: Supplementary file 1 [file ijms-25-06431-s001.zip › ijms-3040051-supplementary.pdf]

SUPPLEMENTARY TABLES

Table S1. Patient characteristics. Given is the number of patients that were assessed for the respective factor (second column) as well as the number and percentage of patients harboring the respective risk factor or absence thereof (last two columns). GPI, gene expression-based proliferation index. Crea, creatinine (≥2mg/dL = high, <2mg/dL = low). (r)ISS, (revised) International Staging System.

Factor	n assessed	Level	n	%
t(4;14)	352		49	13.9
t(11;14)	354		68	19.2
t(14;16)	340		6	1.8
hyperdiploidy	341		175	51.3
1q21+	344	>3	34	9.9
		3	78	22.7
		2	232	67.4
del17p	350		37	10.6
del13q14	354		171	48.3
del13q14 only	273		106	38.8
del8p21	344		80	23.3
GPI	203	high	15	7.4
		medium	111	54.7
		low	77	37.9
crea	395	high	42	10.6
		low	353	89.4
ISS	368	I	148	40.2
		II	129	35.1
		III	91	24.7
rISS	309	III	50	16.2
		II	169	54.7
		I	90	29.1

Table S2. Differential gene expression. Given are the 292 differentially expressed genes between malignant plasma cells of patients responding with ≥nCR by PAD- vs. VAD-based induction treatment followed by high-dose melphalan (see also Figure 6).

	Gene Symbol	Probeset	log Fold Change	P-value
1	*ELOVL4*	219532_at	3.82	0.003
2	*ETV1*	221911_at	3.50	0.019
3	*MSRB3*	225782_at	3.39	0.009
4	*SHISA2*	230493_at	2.98	0.036
5	*SATB1*	203408_s_at	2.87	0.005
6	*PERP*	217744_s_at	2.66	0.012
7	*GPRC5D*	221297_at	2.52	0.020
8	*CD1D*	205789_at	2.43	0.032
9	*PTGS2*	204748_at	2.43	0.027
10	*TIMP2*	203167_at	2.37	0.040
11	*GAREM1*	228115_at	2.35	0.033
12	*SYPL1*	201259_s_at	2.18	0.003
13	*PRR15*	226961_at	2.17	0.021
14	*MAP2*	225540_at	2.15	0.043
15	*MEST*	202016_at	2.13	0.011
16	*VEGFC*	209946_at	2.08	0.018
17	*RALGPS2*	220338_at	2.01	0.006
18	*ETV5*	203349_s_at	1.99	0.006
19	*TRIM2*	202342_s_at	1.98	0.049
20	*MBNL3*	229498_at	1.97	0.018
21	*DAPK1*	203139_at	1.95	0.045
22	*PELI1*	232213_at	1.95	0.013
23	*KRR1*	232441_at	1.92	0.001
24	*RAB8B*	222846_at	1.91	0.005
25	*ZNF711*	228988_at	1.89	0.040
26	*LOC728613*	1569110_x_at	1.87	0.049
27	*AKT3*	212607_at	1.86	0.035
28	*PAWR*	204004_at	1.85	0.008
29	*TBRG1*	226318_at	1.85	0.002
30	*MYLIP*	228097_at	1.84	0.005
31	*TEAD1*	224955_at	1.83	0.047
32	*SKIL*	217591_at	1.81	0.002
33	*LOC401261*	1559964_at	1.79	0.032
34	*FOXP2*	235201_at	1.77	0.017
35	*DENND1B*	1564164_at	1.72	0.033
36	*BMPR1A*	213578_at	1.72	0.047
37	*CCDC50*	236831_at	1.71	0.043
38	*NAP1L5*	228062_at	1.71	0.002
39	*MAP9*	228423_at	1.70	0.03
40	*FAM126A*	227239_at	1.69	0.024
41	*CASD1*	219342_at	1.68	0.037
42	*CXCR4*	209201_x_at	1.68	0.026
43	*PON2*	210830_s_at	1.67	0.018
44	*PRKAR1A*	200604_s_at	1.66	0.012
45	*BAG5*	202984_s_at	1.65	0.005
46	*GRAMD3*	218706_s_at	1.65	0.022
47	*RB1*	203132_at	1.65	0.021
48	*MLK4*	228565_at	1.61	0.028
49	*PFN2*	204992_s_at	1.60	0.019
50	*FAM60A*	223038_s_at	1.58	0.025
51	*PLEKHF2*	218640_s_at	1.58	0.031
52	*ALKBH5*	1553101_a_a	1.57	0.005
53	*NUCKS1*	222027_at	1.55	0.018
54	*FLI1*	210786_s_at	1.54	0.010
55	*PTP4A1*	200730_s_at	1.54	0.028
56	*DCAF8*	243318_at	1.54	0.019
57	*ENPP5*	227803_at	1.53	0.048
58	*LRRFIP1*	201862_s_at	1.52	0.025
59	*GLIDR*	228040_at	1.51	0.037
60	*PMS2P5*	242201_at	1.51	0.011
61	*TRIB2*	202478_at	1.50	0.030
62	*C9orf40*	218904_s_at	1.50	0.047
63	*PEX11B*	202658_at	1.50	0.021
64	*DNAJC6*	204720_s_at	1.48	0.016
65	*SLC26A6*	232551_at	1.48	0.007
66	*VAPA*	228480_at	1.47	0.010
67	*MTDH*	227277_at	1.46	0.040
68	*HNRNPM*	214918_at	1.45	0.011
69	*MSANTD3*	1555841_at	1.45	0.004
70	*KLF6*	208961_s_at	1.45	0.029
71	*PDE7A*	223358_s_at	1.44	0.032
72	*EIF2S3*	205321_at	1.44	0.042
73	*ZFAND6*	239757_at	1.44	0.013
74	*PCNP*	1554868_s_a	1.42	0.0001
75	*VGLL4*	212399_s_at	1.42	0.009
76	*LRRC1*	218816_at	1.42	0.027
77	*ADSS*	221761_at	1.41	0.005
78	*TSC22D2*	204094_s_at	1.41	0.001
79	*ASMTL*	36554_at	1.41	0.015
80	*ACVR1B*	213198_at	1.41	0.002
81	*HMCES*	201677_at	1.40	0.022
82	*PLCL1*	205934_at	1.40	0.015
83	*RSL1D1*	212019_at	1.39	0.021
84	*PRPF39*	220553_s_at	1.38	0.009
85	*RPL15*	240806_at	1.36	0.006
86	*CD46*	211574_s_at	1.36	0.017
87	*TSC22D3*	208763_s_at	1.35	0.004
88	*CNNM3*	220739_s_at	1.35	0.038
89	*TSC22D1*	215111_s_at	1.34	0.034
90	*IFRD1*	230048_at	1.33	0.016
91	*SP3*	232529_at	1.33	0.007
92	*CD48*	237759_at	1.33	0.016
93	*PRKCD*	202545_at	1.32	0.024
94	*RBM14*	204178_s_at	1.31	0.001
95	*MPP6*	205429_s_at	1.31	0.040
96	*JAZF1*	225798_at	1.31	0.032
97	*LOC1019283*	1558795_at	1.30	0.041
98	*SCAF4*	222310_at	1.29	0.047
99	*ZNF207*	229765_at	1.29	0.016
100	*CPNE1*	206918_s_at	1.27	0.004

Table S2ff.

101	*SPINT2*	210715_s_at	1.27	0.024
102	*SKP1*	200719_at	1.27	0.049
103	*TMX1*	208097_s_at	1.26	0.038
104	*LOC100506990*	227917_at	1.26	0.045
105	*VPS37B*	221704_s_at	1.25	0.012
106	*APOOL*	235433_at	1.24	0.029
107	*ZDBF2*	228749_at	1.23	0.019
108	*SMG7*	201794_s_at	1.22	0.025
109	*PRPF38B*	230270_at	1.22	0.017
110	*SMARCC1*	201075_s_at	1.21	0.046
111	*PSMA3-AS1*	225724_at	1.20	0.017
112	*CBX3*	230998_at	1.20	0.012
113	*ING1*	244177_at	1.19	0.021
114	*GCC1*	218912_at	1.17	0.005
115	*NUP50*	218294_s_at	1.16	0.009
116	*CTDSPL2*	223271_s_at	1.16	0.007
117	*LOC100996579*	236451_at	1.16	0.025
118	*CDKAL1*	214877_at	1.16	0.032
119	*ANP32A*	201051_at	1.16	0.002
120	*UBA6*	218340_s_at	1.15	0.047
121	*SERBP1*	227369_at	1.15	0.017
122	*ARIH1*	201879_at	1.14	0.029
123	*SRSF6*	206108_s_at	1.14	0.043
124	*UGT2B15*	207392_x_at	1.13	0.047
125	*FCF1*	215567_at	1.12	0.030
126	*CDKN2AIP*	218929_at	1.12	0.002
127	*FGD2*	1553906_s_at	1.12	0.033
128	*TMEM192*	226589_at	1.10	0.015
129	*NSD1*	235760_at	1.10	0.039
130	*CNOT7*	233019_at	1.08	0.034
131	*PDSS1*	220865_s_at	1.07	0.032
132	*HIF1AN*	226648_at	1.07	0.035
133	*ZNF551*	211721_s_at	1.06	0.040
134	*SND1-IT1*	210109_at	1.06	0.012
135	*RBBP5*	205169_at	1.05	0.020
136	*ARFGAP2*	211975_at	1.04	0.033
137	*SP1*	224754_at	1.03	0.047
138	*UBXN7*	217100_s_at	1.03	0.018
139	*RBM48*	221595_at	1.03	0.045
140	*PEX5*	203244_at	1.02	0.002
141	*DIS3*	218362_s_at	1.02	0.040
142	*LINC00888*	228275_at	1.02	0.009
143	*HDGF*	200896_x_at	1.02	0.007
144	*ACLY*	210337_s_at	1.02	0.030
145	*TNFRSF10A*	231775_at	1.02	0.031
146	*DPF2*	202116_at	1.01	0.023
147	*SLC25A36*	201918_at	1.01	0.012
148	*GLUD2*	215794_x_at	1.01	0.029
149	*TNS1*	221748_s_at	-1.00	0.002
150	*NPL*	223405_at	-1.02	0.036

151	*PMP22*	210139_s_at	-1.02	0.004
152	*ARHGEF26*	227197_at	-1.02	0.040
153	*CBL*	229010_at	-1.03	0.036
154	*RRAD*	204803_s_at	-1.03	0.019
155	*LRRC8C*	228314_at	-1.05	0.030
156	*CRYL1*	220753_s_at	-1.00	0.037
157	*BCAR3*	204032_at	-1.06	0.035
158	*FPR3*	230422_at	-1.07	0.026
159	*TK2*	204276_at	-1.09	0.024
160	*SYTL3*	238423_at	-1.09	0.006
161	*EPB41L3*	212681_at	-1.10	0.036
162	*ADRB1*	229309_at	-1.10	0.044
163	*NRP1*	212298_at	-1.10	0.035
164	*AAMDC*	221599_at	-1.10	0.028
165	*CCNYL1*	227280_s_at	-1.10	0.049
166	*PID1*	219093_at	-1.10	0.016
167	*CWC22*	226588_at	-1.11	0.046
168	*SLC31A1*	203971_at	-1.12	0.002
169	*FYB*	211795_s_at	-1.12	0.023
170	*B9D1*	210534_s_at	-1.12	0.031
171	*BLVRB*	202201_at	-1.13	0.047
172	*CYP4X1*	227702_at	-1.13	0.003
173	*IGLL3P*	215946_x_at	-1.14	0.030
174	*DYNLT1*	201999_s_at	-1.14	0.016
175	*GPR34*	223620_at	-1.14	0.039
176	*NBAS*	242049_s_at	-1.17	0.022
177	*C17orf58*	226901_at	-1.17	0.007
178	*SWI5*	226027_at	-1.18	0.012
179	*RASSF4*	226436_at	-1.18	0.028
180	*ITGB5*	201125_s_at	-1.18	0.022
181	*MARCH3*	213256_at	-1.18	0.042
182	*IGLV@*	216566_at	-1.19	0.018
183	*SPIC*	1553851_at	-1.20	0.019
184	*PTGER2*	206631_at	-1.20	0.001
185	*TMEM37*	227190_at	-1.21	0.017
186	*MEF2A*	208328_s_at	-1.22	0.024
187	*SPIRE1*	224995_at	-1.22	0.046
188	*ZNF195*	204234_s_at	-1.23	0.025
189	*GALNS*	206335_at	-1.23	0.013
190	*SPATS2L*	222154_s_at	-1.26	0.014
191	*TCN2*	204043_at	-1.26	0.025
192	*IL18*	206295_at	-1.27	0.011
193	*SCNN1B*	205464_at	-1.28	0.012
194	*FAM213B*	231835_at	-1.29	0.021
195	*ODF3B*	238327_at	-1.29	0.006
196	*CXCL9*	203915_at	-1.29	0.009
197	*ADAMTSL3*	213974_at	-1.29	0.006
198	*EFEMP2*	206580_s_at	-1.30	0.005
199	*RAB20*	219622_at	-1.30	0.048
200	*KCNAB1*	210078_s_at	-1.30	0.013

Table S2ff.

201	*CDH19*	206898_at	-1.30	0.027
202	*SASH1*	226022_at	-1.32	0.003
203	*VAMP5*	204929_s_at	-1.33	0.034
204	*SOBP*	218974_at	-1.33	0.005
205	*IFITM10*	227863_at	-1.33	0.018
206	*ACTN1*	208636_at	-1.34	0.019
207	*SGTB*	228745_at	-1.34	0.038
208	*GDPD1*	238681_at	-1.35	0.005
209	*ADORA3*	223660_at	-1.36	0.008
210	*AMPD1*	206121_at	-1.36	0.033
211	*SRP72*	208803_s_at	-1.37	0.030
212	*CCL2*	216598_s_at	-1.37	0.001
213	*ADAP2*	219358_s_at	-1.37	0.002
214	*IKBIP*	227295_at	-1.38	0.029
215	*CERCAM*	224794_s_at	-1.38	0.046
216	*LARS*	223888_s_at	-1.38	0.039
217	*FRMD4B*	213056_at	-1.40	0.032
218	*EMC3*	228775_at	-1.40	0.001
219	*HBE1*	205919_at	-1.41	0.018
220	*HS3ST1*	205466_s_at	-1.42	0.004
221	*PLA2G7*	206214_at	-1.42	0.042
222	*UGGT2*	235749_at	-1.43	0.036
223	*SLA*	203761_at	-1.44	0.007
224	*TXLNB*	227834_at	-1.47	0.041
225	*RNFT2*	221908_at	-1.48	0.029
226	*SIKE1*	221705_s_at	-1.49	0.039
227	*SLCO2B1*	203473_at	-1.49	0.050
228	*GAPVD1*	212804_s_at	-1.50	0.033
229	*PEX3*	203970_s_at	-1.50	0.022
230	*TMEM98*	223170_at	-1.50	0.006
231	*ASPH*	224996_at	-1.54	0.011
232	*GLIPR1*	226142_at	-1.54	0.039
233	*LOC100506100*	228773_at	-1.55	0.006
234	*CEBPD*	203973_s_at	-1.57	0.025
235	*MT1F*	213629_x_at	-1.57	0.007
236	*ANKRD36*	214723_x_at	-1.58	0.048
237	*IQCG*	221185_s_at	-1.59	0.036
238	*RRBP1*	201206_s_at	-1.60	0.031
239	*P2RY6*	208373_s_at	-1.60	0.029
240	*HMOX1*	203665_at	-1.62	0.042
241	*IGK*	211908_x_at	-1.65	0.017
242	*TMX3*	1552822_at	-1.66	0.009
243	*REPS2*	227425_at	-1.66	0.031
244	*IGLJ3*	211798_x_at	-1.67	0.017
245	*CCL18*	209924_at	-1.68	0.014
246	*GCAT*	205164_at	-1.71	0.009
247	*CCL8*	214038_at	-1.73	0.015
248	*ENAM*	223893_at	-1.73	0.015
249	*GBP1*	202270_at	-1.74	0.025
250	*RNASE2*	206111_at	-1.75	0.004

251	*EID3*	231292_at	-1.76	0.018
252	*GPR183*	205419_at	-1.76	0.001
253	*SLC1A3*	202800_at	-1.76	0.006
254	*RUFY3*	229334_at	-1.78	0.005
255	*SMPDL3B*	205309_at	-1.80	0.012
256	*CFAP36*	224968_at	-1.81	0.002
257	*FAS*	204780_s_at	-1.85	0.025
258	*GPNMB*	201141_at	-1.86	0.011
259	*LINC01419*	1559213_at	-1.89	0.001
260	*RGL1*	209568_s_at	-1.89	0.006
261	*IGSF6*	206420_at	-1.92	0.013
262	*AXL*	202686_s_at	-1.92	0.019
263	*PLCB4*	203895_at	-1.93	0.003
264	*BVES*	228783_at	-1.94	0.038
265	*B3GALNT1*	211379_x_at	-1.96	0.033
266	*TIMD4*	1552280_at	-1.98	0.012
267	*IGHD*	214973_x_at	-2.01	0.010
268	*SLC22A4*	205896_at	-2.02	0.001
269	*PPP1R16B*	41577_at	-2.05	0.012
270	*IL23R*	1552912_a_a	-2.06	0.003
271	*SLC40A1*	223044_at	-2.08	0.032
272	*FGL2*	204834_at	-2.10	0.019
273	*CCR1*	205098_at	-2.13	0.031
274	*POPDC3*	219926_at	-2.14	0.011
275	*HLA-DQA1*	213831_at	-2.16	0.020
276	*KDELR3*	207264_at	-2.18	0.014
277	*CD69*	209795_at	-2.18	0.033
278	*CDKN2C*	204159_at	-2.36	0.004
279	*PDK4*	225207_at	-2.46	0.025
280	*CDC27*	217878_s_at	-2.52	0.016
281	*TGFBI*	201506_at	-2.57	0.007
282	*BMS1P20*	224342_x_at	-2.57	0.013
283	*RUNX2*	232231_at	-2.61	0.042
284	*TLR7*	220146_at	-2.66	0.004
285	*MOCOS*	219959_at	-2.72	0.034
286	*INHBE*	210587_at	-2.73	0.005
287	*IGLV1-44*	217227_x_at	-2.94	0.001
288	*ICAM4*	207194_s_at	-3.01	0.004
289	*NID2*	204114_at	-3.17	0.013
290	*FSTL5*	232010_at	-3.18	0.001
291	*ATP10B*	214070_s_at	-3.96	0.004
292	*DCLK1*	205399_at	-4.33	0.006
